# A study on the spatial distribution of life expectancy and its air pollution factors in China based on geographically weighted regression

**DOI:** 10.3389/fpubh.2025.1565744

**Published:** 2025-04-28

**Authors:** Ke Hu, Xing Zhang, Xingjin Yang, Mingyang Yu

**Affiliations:** ^1^Xiamen Haicang Hospital, Xiamen, China; ^2^Nanjing Lishui Dongping Street Health Center, Nanjing, China; ^3^QianDongNanZhou Center for Disease Control and Prevention, QianDongNanZhou, China; ^4^Fuwai Central China Cardiovascular Hospital, Zhengzhou, China

**Keywords:** life expectancy, air pollution, spatial heterogeneity, spatial autocorrelation, geographically weighted regression

## Abstract

**Background:**

Life expectancy in China has demonstrated a consistent upward trend, yet significant disparities persist across provinces. Addressing these regional imbalances necessitates a comprehensive investigation into the determinants of life expectancy. Previous research has largely overlooked the critical role of spatial heterogeneity, which is essential for understanding the underlying mechanisms driving these disparities. By incorporating spatial analysis, this study aims to identify and address the factors contributing to the uneven distribution of life expectancy across China, thereby providing a more nuanced understanding of regional health inequalities.

**Methods:**

Therefore, this study investigated the spatial distribution characteristics and patterns of life expectancy across 31 provinces in China in 2020 by conducting descriptive and spatial autocorrelation analyses, utilizing life expectancy data alongside key air pollution indicators (PM_2.5_, SO_2_, NO_2_, and PM_10_). To address spatial heterogeneity, the geographically weighted regression (GWR) model was applied to assess the regional variations in the impact of air pollutants on life expectancy. This approach allows for the incorporation of geographic coordinates into the regression coefficients, capturing localized effects and providing a more nuanced understanding of the relationship between air pollution and life expectancy across different regions.

**Results:**

The findings revealed that in 2020, life expectancy in China exhibited a distinct east-to-west decreasing trend, demonstrating significant spatial autocorrelation that was predominantly characterized by two aggregation patterns: high-high and low-low clusters. The analysis demonstrated that air pollutants, including SO_2_, NO_2_, and PM_10_, exerted significant influences on life expectancy, albeit with regional variations. Specifically, SO_2_ exhibited a more pronounced negative impact on life expectancy in southern cities, while NO_2_ demonstrated a stronger effect in northwestern regions. Notably, PM_10_ showed a significant influence limited to Yunnan Province, highlighting the spatial heterogeneity in the relationship between air pollution and life expectancy across China.

**Conclusion:**

These findings highlight the imperative for local governments to develop and implement region-specific air pollution control measures, taking into account the unique environmental and socio-economic conditions of their respective areas.

## 1 Introduction

With sustained economic development and advancements in medical care, life expectancy in China has demonstrated a consistent upward trajectory (1–3). Specifically, between 2010 and 2020, the average life expectancy increased from 74.83 to 77.90 years. However, significant regional disparities persist across provinces (4). In 2020, Shanghai recorded the highest life expectancy at 82.55 years, while Tibet had the lowest at 72.19 years, reflecting a notable gap of nearly 10 years. These pronounced variations underscore the necessity of a comprehensive investigation into the determinants of life expectancy in China. Such an exploration is crucial to identifying effective strategies for addressing the disparities in life expectancy between regions.

Numerous studies have established that air pollution significantly impacts life expectancy (5). Exposure to ambient air pollutants, including sulfur dioxide (SO_2_), fine particulate matter (PM_2.5_), and nitrogen oxides (NO_x_), was strongly associated with an elevated risk of various diseases and a marked reduction in life expectancy (6–13). For instance, Wang et al. highlighted that pollutants such as PM_10_ and SO_2_ exerted a detrimental effect on life expectancy (14). Specifically, PM_10_ had been identified as a critical contributor to respiratory diseases, cardiovascular conditions, and hypertension. The long-term exposure of populations to air pollution has been shown to substantially diminish life expectancy. This relationship was particularly evident with SO_2_, where a 10 ug/m^3^ increase in concentration had been linked to a 0.35-year reduction in life expectancy (14). These findings underscored the profound public health implications of air pollution and the urgent need for effective mitigation strategies.

In the realm of methodologies for investigating the determinants of life expectancy, earlier studies predominantly utilized regression analyses that overlooked spatial information, such as multiple linear regression. However, these models often suffer from multicollinearity among independent variables and fail to incorporate spatial data (4), leading to suboptimal model fit and residuals that exhibit spatial autocorrelation. In response, recent research has increasingly adopted spatial regression models, including the spatial error model and the spatial lag model (15), to better capture spatial dependencies in life expectancy studies. While these global spatial regression models account for spatial correlations among variables, they do not address the spatial heterogeneity—the varying impact of each factor on life expectancy across different geographic locations. To address this limitation, the geographically weighted regression (GWR) model has been introduced as a local regression approach. GWR integrates the geographic locations of independent variables into the regression coefficients, thereby elucidating how the influence of various factors on life expectancy varies spatially (16, 17).

Our study comprehensively utilize the GWR model to analyze the impact of air pollution on life expectancy in China. Compared to traditional global regression models, the GWR model constructs local regression equations at each spatial location, taking into account the spatial heterogeneity. This allows it to more accurately reflect regional differences in the impact of air pollution on life expectancy. Based on the analysis results of the GWR model, policymakers can more accurately identify regions where air pollution has a significant impact on life expectancy and implement more targeted governance measures in these areas.

Consequently, this study investigated the spatial distribution characteristics and patterns of life expectancy by employing descriptive and spatial autocorrelation analyses. Subsequently, we utilized the GWR model to examine the impact of air pollution factors on life expectancy across 31 regions in China during 2020. The findings offer a robust scientific foundation for optimizing the allocation of healthcare resources and informing region-specific policy formulation.

## 2 Data and methods

### 2.1 Variable selection and data sources

To comprehensively investigate the impact of air pollution on life expectancy across Chinese provinces, we selected key variables—PM_2.5_, SO_2_, NO_2_, and PM_10_—based on their established relevance to life expectancy in prior literature and data availability. The study was conducted at the provincial administrative level, encompassing all 31 mainland Chinese provinces (excluding Hong Kong, Macao, and Taiwan). Life expectancy data for 2020 were sourced from the China Health Statistical Yearbook, while air pollution data (PM_2.5_, SO_2_, NO_2_, and PM_10_) were obtained from regional ecological and environmental bulletins.

### 2.2 Statistical methods

#### 2.2.1 Descriptive analyses

The spatial distribution characteristics of life expectancy and air pollution factors across the 31 districts were visualized using thematic maps.

#### 2.2.2 Spatial autocorrelation analysis

This study examined spatial autocorrelation in life expectancy at both global and local scales. At the global level, we employed Global Moran’s I, a statistical index ranging from −1 to 1. Values greater than 0 indicate positive spatial autocorrelation, reflecting clustering of life expectancy; values closer to 0 suggest a random spatial distribution, implying no autocorrelation; and values less than 0 denote negative spatial autocorrelation, signifying a dispersed distribution. The mathematical formulation of Global Moran’s I is as follows (18) (Equation 1):


(1) $$I=\frac{n\overset{n}{\underset{i=1}{\sum }}\overset{n}{\underset{j=1}{\sum }}W_{ij}\left(x_{i}-\bar{x}\right)\left(x_{j}-\bar{x}\right)}{\overset{n}{\underset{i=1}{\sum }}\overset{n}{\underset{j=1}{\sum }}W_{ij}\overset{n}{\underset{i=1}{\sum }}{\left(x_{i}-\bar{x}\right)}^{2}}$$

where *x*_i_ and *x*_j_ represent the life expectancy of the *i*-th region and *j*-th regions, respectively; $\bar{x}$ denotes the mean life expectancy; *W*_ij_ is the spatial weight matrix based on contiguity edges corner; n refers to the number of spatial units.The statistical significance of Moran’s I was tested using the Z statistic(Equation 2):


(2) $$Z=\frac{I-E\left(I\right)}{\sqrt{var\left(I\right)}}$$

where E(*I*) and var.(*I*) represent the expected value and variance of Moran’s I, respectively. At a significance level of 0.05, Z > 1.96 (i.e., *p* < 0.05) indicates spatial autocorrelation in life expectancy.

At the local level, we utilized Local Moran’s I (LISA) to identify spatial aggregation patterns (e.g., high-high, low-low, high-low, and low-high) in life expectancy across provinces. The calculation formula (Equation 3) for LISA is analogous to that of Global Moran’s I, and the spatial aggregation patterns were visualized using LISA cluster maps.


(3) $$I_{i}=\frac{n\left(x_{i}-\bar{x}\right)\overset{n}{\underset{j=1}{\sum }}W_{ij}\left(n_{j}-\bar{n}\right)}{\overset{n}{\underset{j=1}{\sum }}{\left(x_{j}-\bar{x}\right)}^{2}}$$

#### 2.2.3 GWR model

Multicollinearity testing is a critical step in data analysis to ensure the reliability of regression models. In this study, the Variance Inflation Factor (VIF) and the condition number were employed to evaluate multicollinearity among the variables (19). For global multicollinearity assessment, a widely accepted threshold is a VIF value exceeding 7.5, which indicates severe multicollinearity. Variables with VIF values above this threshold are excluded from further analysis to mitigate potential biases.

Additionally, local multicollinearity can introduce instability in GWR estimates (20). The condition number is used to detect local multicollinearity, with values greater than 30 in a specific region suggesting potential unreliability in the model’s fitting results for that area.

##### 2.2.3.1 GWR model setting

The GWR model extends the traditional multiple linear regression framework by incorporating the geographic coordinates of sample data into the regression coefficients, thereby capturing spatial heterogeneity. The general form of the GWR model is expressed as follows (Equation 4):


(4) $$Q_{i}=\beta_{0}\left(u_{i},v_{i}\right)+\overset{m}{\underset{k=1}{\sum }}\beta_{k}\left(u_{i},v_{i}\right)x_{ik}+\varepsilon_{i}\left(i=1,2\ldots 31\right)$$

In this study, *Q*_i_ is used to denote the life expectancy of the *i*-th province, while *β*_0_ is employed to represent the regression constant for the *i*-th province. The term (*u*_i_,*v*_i_) denotes the geographic location of the *i*-th province, which is determined using the center of mass coordinates of each province in the GWR model. Finally, *β_k_*(*u_i_,v_i_*)is used to represent the regression coefficient of the *k*-th province independent variable for the *i*-th province, *x*_ik_ represents the observed value of the independent variable *x*_k_ in the *i*-th province, and $\varepsilon_{i}$ represents the error term for the *i*-th province (21, 22), where $\varepsilon_{i}\sim N\left(0,\sigma^{2}\right)$.

Since the regression coefficients in the GWR model vary across provinces, a parameter estimation method was proposed based on Weighted Least Squares (WLS). This approach assigns higher weights to observations that are geographically closer to the target location and lower weights to those that are farther away, reflecting the principle of spatial proximity. For a detailed mathematical representation, refer to Equation 5:


(5) $$\hat{\beta }\left(u_{i},v_{i}\right)={\left(X^{T}W\left(u_{i},v_{i}\right)X\right)}^{-1}X^{T}W\left(u_{i},v_{i}\right)Q$$

In this formulation, *W*(*u*_i_, *v*_i_) is the spatial weight matrix, *X*^T^ W(*u*_i_, *v*_i_)*X* is the geographically weighted variance–covariance matrix, and *Q* is the vector of dependent variables.

The determination of spatial weights is fundamentally dependent on the choice of the spatial kernel function (23). In this study, the fixed Gaussian function was selected as the spatial kernel function due to its ability to generate a smoother kernel surface and mitigate the risk of data sparsity during the GWR model fitting process. The mathematical expression of the fixed Gaussian function is provided in Equation 6:


(6) $$W_{j}\left(u_{i},v_{i}\right)=\exp{\left(-d_{ij}/\theta \right)}^{2}$$

In this equation, *i* denotes a regression point, *j* denotes a data point, *d*_ij_ represents the Euclidian distance between the two points of *i* and *j*, and *θ* is the bandwidth, which is employed to describe the non-negative decay parameter between the distance and the weights.

The selection of bandwidth plays a critical role in the application of spatial kernel functions, as it directly influences the accuracy and performance of the model. Among the widely used bandwidth optimization techniques, the Corrected Akaike Information Criterion (AIC_c_) is particularly prominent due to its effectiveness in balancing model fit and complexity. The mathematical formulation of the AIC_c_ criterion is provided in Equation 7:


(7) $$\mathrm{AI}C_{c}=2n\log_{e}\left(\hat{\sigma }\right)+2n\log_{e}\left(2\pi \right)+n\left(\frac{n+tr\left(S\right)}{n-2-tr\left(S\right)}\right)$$

In this equation, *n* denotes the number of sample points, $\hat{\sigma }$is the standard deviation of the residual term, and *tr*(S) is the trajectory of the spatial weight matrix. Specifically, the bandwidth associated with the minimum AIC_c_ value is identified as the optimal choice.

##### 2.2.3.2 Evaluation of model fitting effectiveness

To assess the goodness-of-fit of the GWR model, this study employed the Multiple Linear Regression (MLR) model as a benchmark for comparative performance evaluation. The MLR model was constructed using the same dataset to ensure consistency in the comparative analysis. The mathematical formulation of the MLR model is presented in Equation 8:


(8) $$Q_{i}=\beta_{0}+\underset{a}{\sum }\beta_{a}x_{\mathrm{ai}}+\varepsilon_{i}$$

The dependent variable *Q*_i_ denotes the life expectancy of the *i*-th province, while *β*_0_ represents a constant term. *x*_ai_ denotes the *a*-th independent variable of the *i*-th province, *β*_a_ denotes the coefficient of the *a*-th independent variable, and the residuals are assumed to be normally distributed.

To evaluate the goodness-of-fit of the GWR model, this study employed three key metrics: R^2^, Adjusted R^2^, and the Corrected Akaike Information Criterion (AIC_c_), with the MLR model serving as a benchmark for comparison. It was hypothesized that higher values of R^2^ and Adjusted R^2^ indicate a greater proportion of variance explained by the regression equation, thereby reflecting a better model fit.

Furthermore, the AIC_c_ serves a dual purpose: it not only facilitates the determination of the optimal bandwidth in GWR but also provides a robust criterion for comparing model superiority. Generally, models with lower AIC_c_ values are considered to exhibit superior fit, as they achieve a better balance between model complexity and explanatory power (22).

#### 2.2.4 Analysis software

The MLR model, GWR model, collinearity diagnostics, spatial autocorrelation analysis, and result visualization were performed using ArcGIS 10.2 software (ESRI, Redlands, CA, United States). The electronic maps utilized for spatial analysis and factor characterization were sourced from the National Geomatics Center of China, which provides authoritative geographic information services. In this study, statistical significance was determined using a two-tailed test with a threshold of *p* < 0.05.

## 3 Results

### 3.1 Spatial distribution characteristics and patterns of life expectancy in China

**Figure 1:**
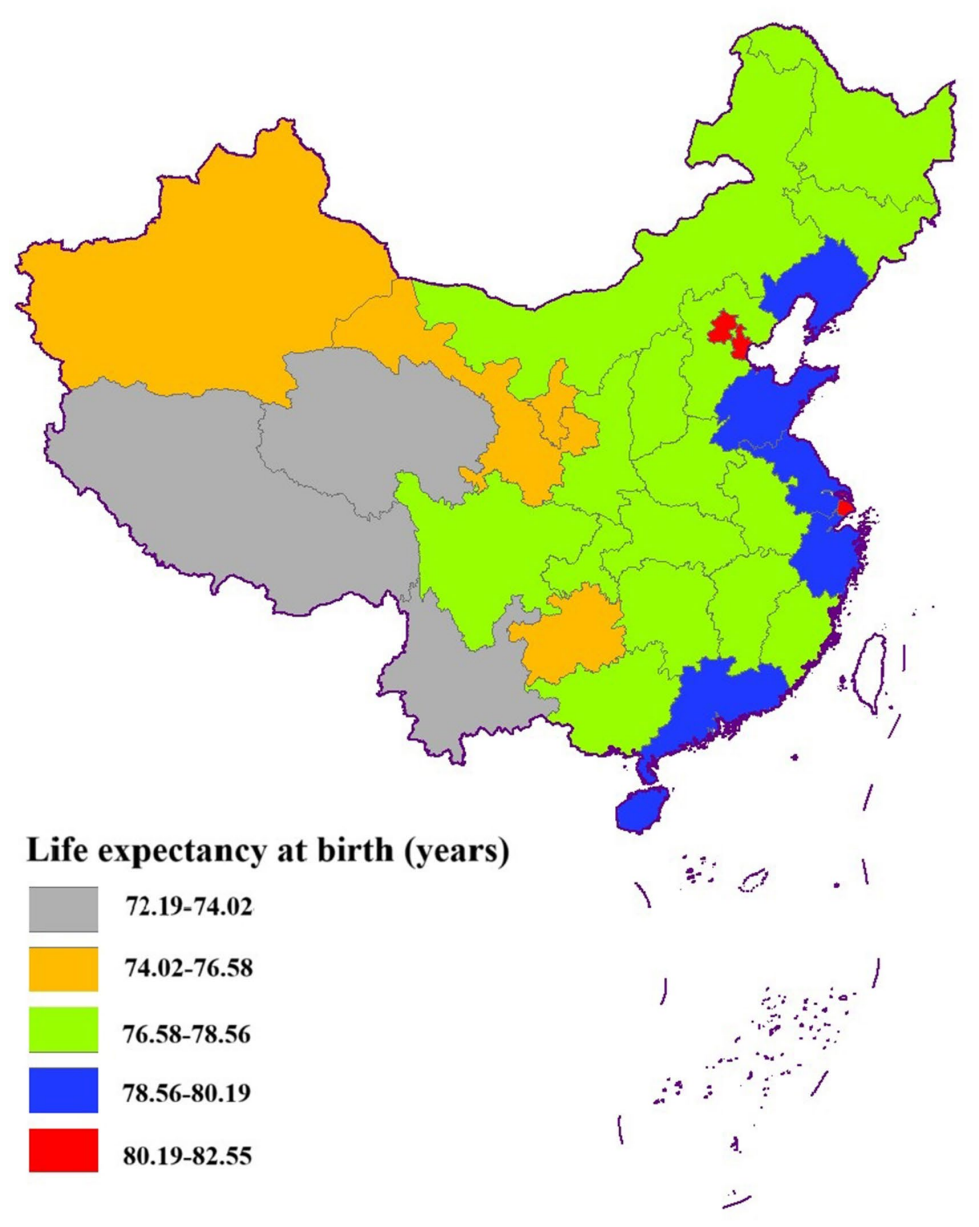
Distribution of life expectancy by province in China in 2020.

In 2020, the average life expectancy in China was 77.90 years. Utilizing the geographic map of mainland China, we generated a spatial distribution map of life expectancy (Figure 1). The analysis revealed a distinct east-to-west decreasing gradient in life expectancy, with the eastern region exhibiting values exceeding 78 years. Notably, Shanghai, Beijing, and Tianjin emerged as the regions with the highest life expectancy, each surpassing 80 years. In contrast, the central region demonstrated life expectancy values ranging between 76 and 78 years. The western region, encompassing Qinghai, Xinjiang, and 10 other provinces, consistently recorded life expectancy values below the national average. Among these, Tibet had the lowest life expectancy at 72.19 years, closely followed by Qinghai at 73.96 years.

**Figure 2:**
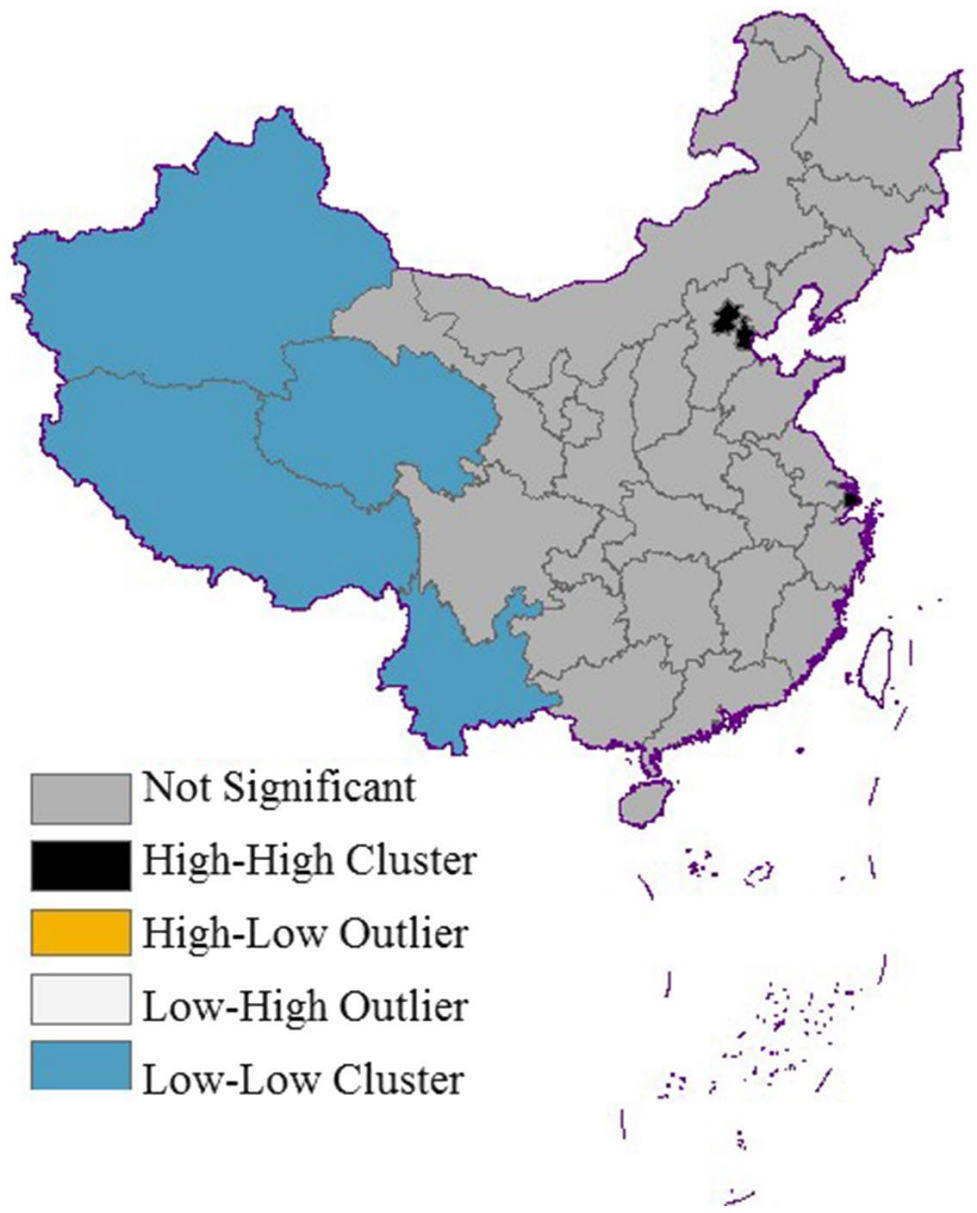
LISA aggregation of life expectancy by province.

The global Moran’s I index was calculated to be 0.534 (*p* < 0.001), demonstrating significant spatial autocorrelation in life expectancy across China. To further investigate the spatial clustering patterns at the provincial level, we generated LISA cluster maps (Figure 2). The Lisa analysis revealed two dominant spatial clustering patterns: high-high and low-low clusters. The high-high clusters were predominantly concentrated in the economically developed eastern coastal regions, notably encompassing Beijing, Tianjin, and Shanghai, which consistently exhibit life expectancy values exceeding 80 years. Conversely, the low-low clusters were primarily located in the western and southwestern provinces, including Xinjiang, Tibet, Yunnan, and Qinghai.

### 3.2 Spatial distribution of air pollution factors

**Figure 3:**
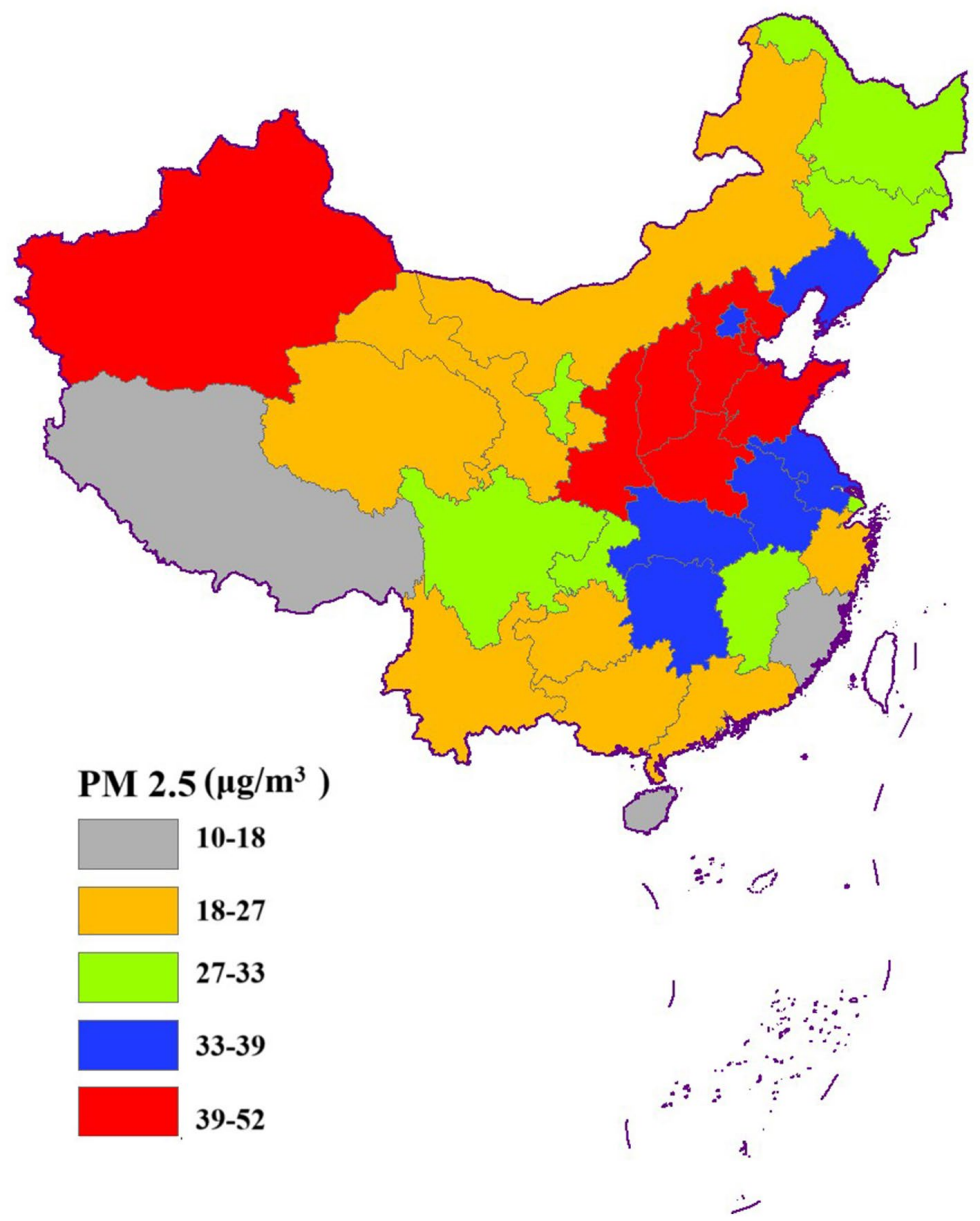
Spatial distribution of PM_2.5_.

**Figure 4:**
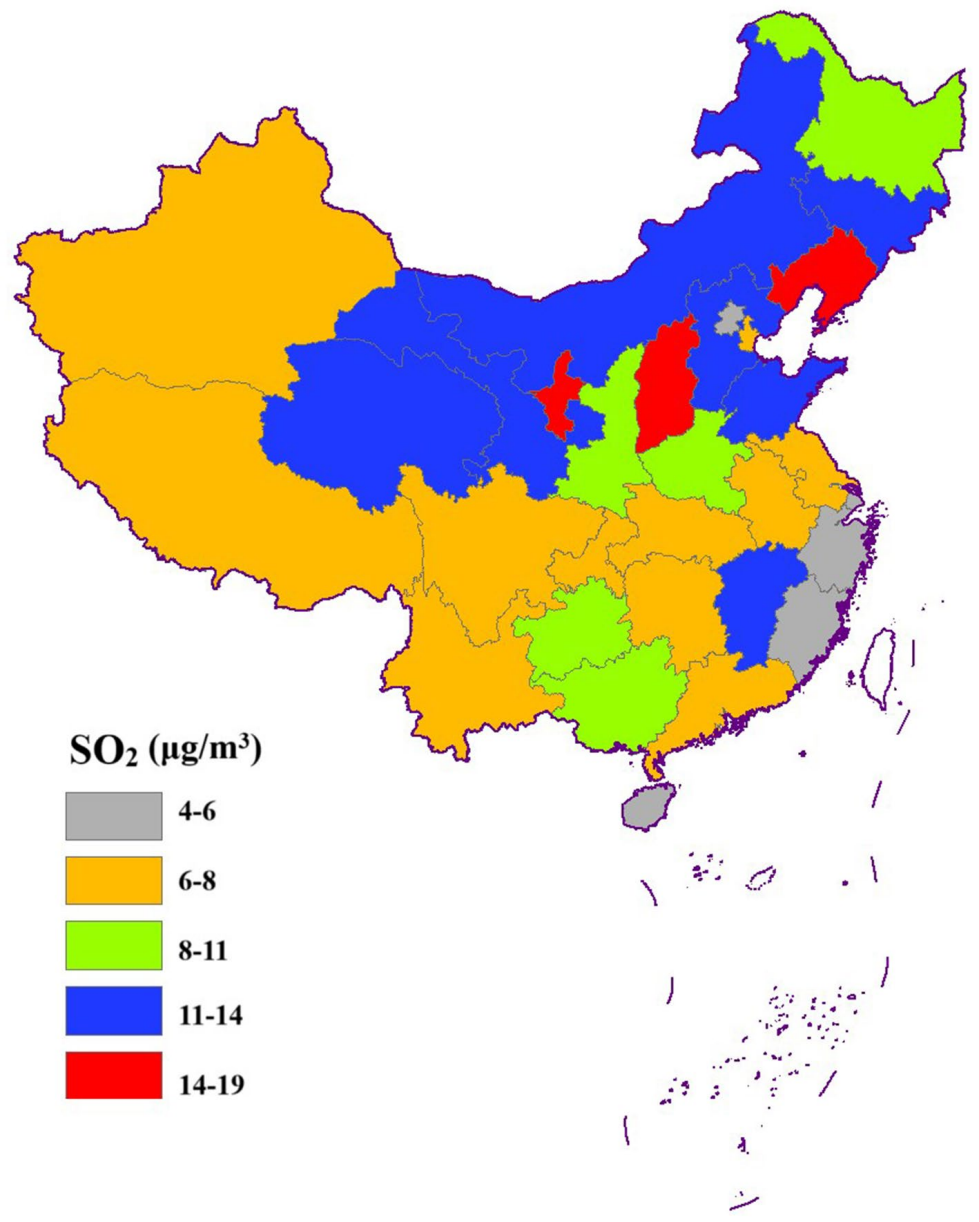
Spatial distribution of SO_2_.

**Figure 5:**
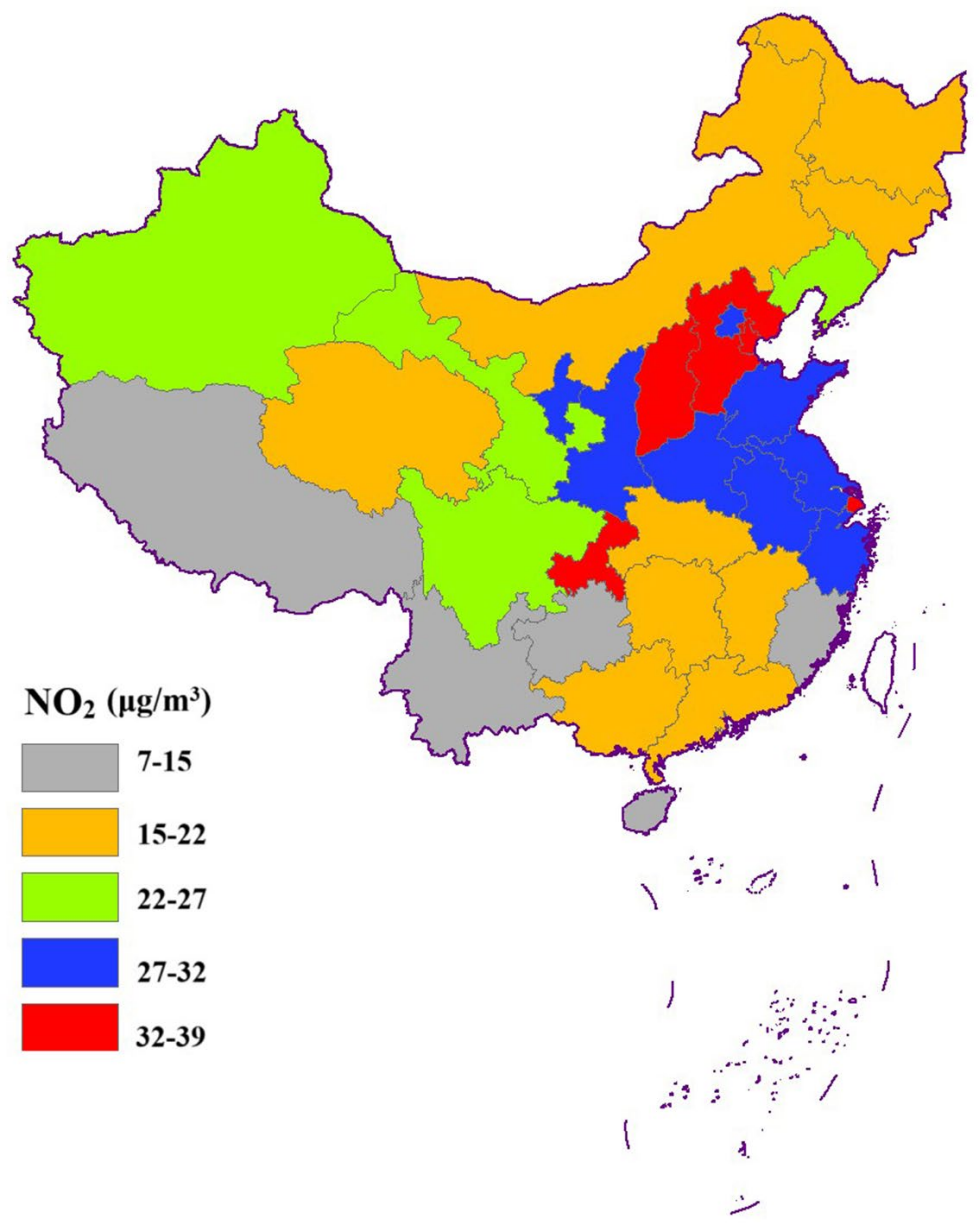
Spatial distribution of NO_2_.

**Figure 6:**
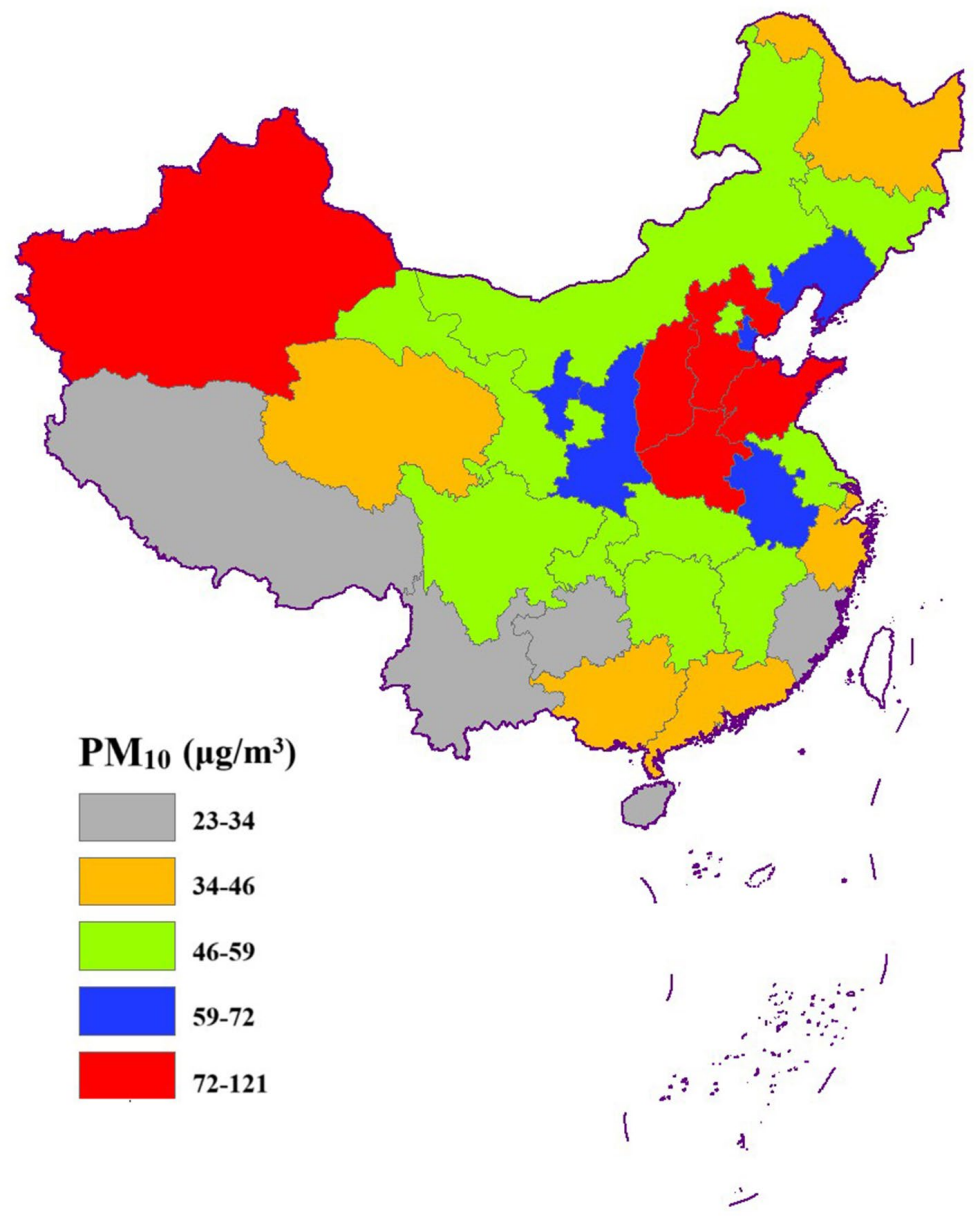
Spatial distribution of PM_10_.

Figures 3–6 illustrate significant spatial variability in the distribution of the four air pollution factors across China. A distinct east-to-west decreasing gradient in air pollution intensity was observed, with the most severe pollution levels concentrated in North China, where industrial activities and urbanization are most intensive. However, Xinjiang exhibited an exception to this trend, with PM_2.5_ and PM_10_ concentrations surpassing those in most other provinces. This anomaly might be attributed to local factors such as desert dust emissions, industrial activities, and unique meteorological conditions.

### 3.3 GWR model results

#### 3.3.1 Variable multicollinearity test

In this study, VIF values were employed to assess the presence of global multicollinearity among the four variables under investigation. The results of this analysis are summarized in Table 1. The VIF value for PM_2.5_ exceeded 7.5, suggesting significant collinearity with other variables. Consequently, PM_2.5_ was excluded from the GWR model to mitigate the impact of multicollinearity on the model’s accuracy and stability.

**Table 1:** Results of multicollinearity test for each variable.

Factors	VIF
PM_2.5_	8.31
SO_2_	1.17
NO_2_	2.89
PM_10_	5.28

#### 3.3.2 GWR model results

##### 3.3.2.1 Effect of SO_2_ on life expectancy

The coefficient values of SO_2_ exhibited a range from −0.18 to −0.42, indicating a consistent negative association with life expectancy. Statistical analysis revealed that the impact of SO_2_ on life expectancy was significant across the majority of cities studied. However, this relationship was not statistically significant in Xinjiang, Tibet, Heilongjiang, and Jilin, suggesting potential regional variations in the effects of SO_2_ exposure. This lack of significance in these regions may be attributed to several factors. Firstly, the relatively low population density and sparse industrial distribution in these areas could result in less exposure to SO_2_ pollution, diminishing its detectable impact on life expectancy (24). Additionally, unique geographical and climatic conditions, such as the high-altitude environment in Tibet or the cold climate in Heilongjiang and Jilin, might influence the dispersion and accumulation of pollutants, thereby altering the observed effects (6). Moreover, the distinct socio-economic structures and varying levels of environmental regulations in these regions could also play a role in mitigating the influence of SO_2_ on life expectancy (25).

**Figure 7:**
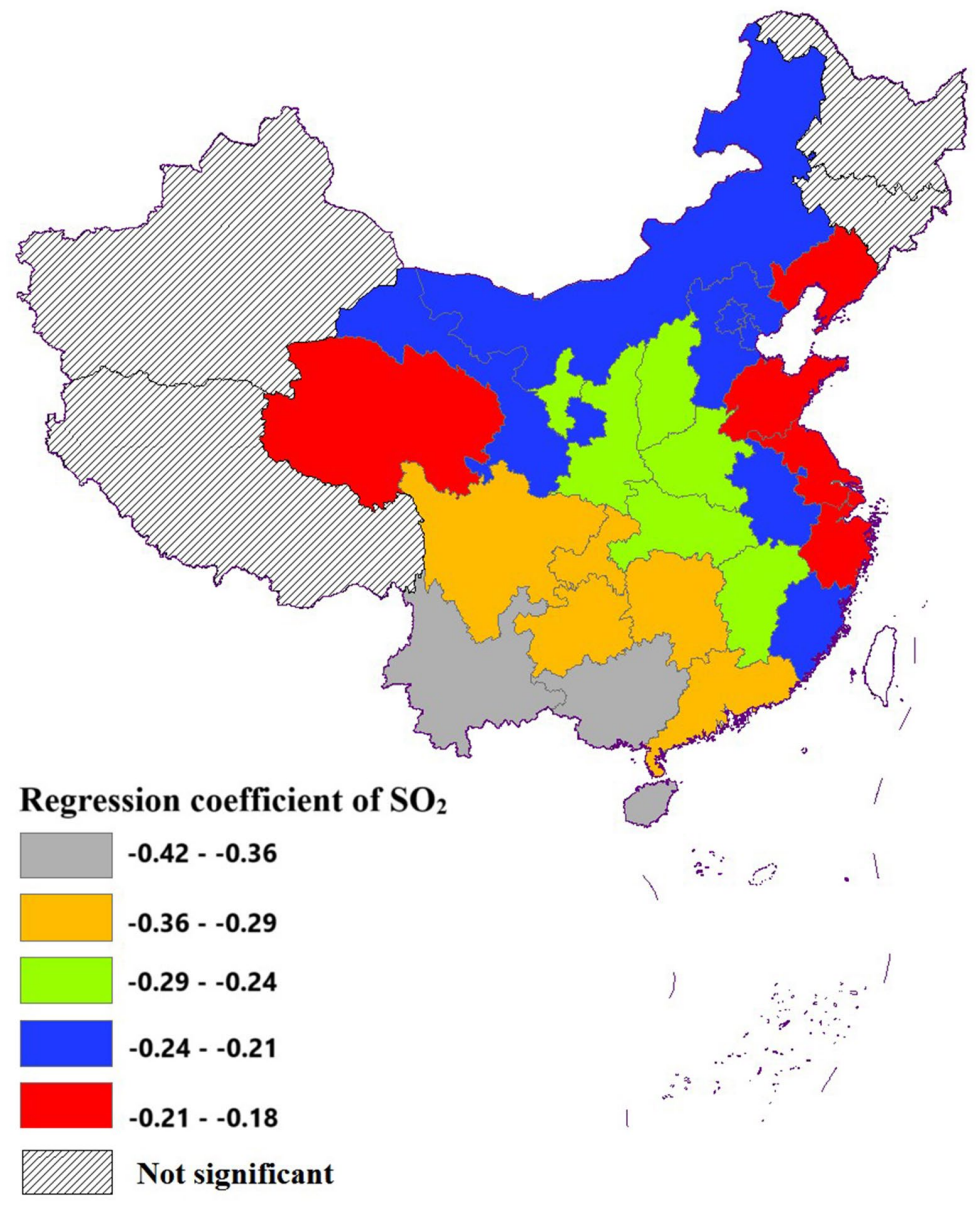
Spatial distribution of the effect of SO_2_ on life expectancy.

The spatial distribution of the regression coefficients for SO_2_ exhibited a pronounced south-to-north gradient, with values progressively decreasing from southern to northern regions. Notably, the impact of SO_2_ on life expectancy was relatively modest in eastern cities, suggesting regional variations in the pollutant’s influence on health outcomes (Figure 7).

##### 3.3.2.2 Effect of NO_2_ on life expectancy

The regression coefficients for NO_2_ exhibited a range from 0.094 to 0.33, indicating a consistent positive association with life expectancy across the majority of regions studied. Statistical analysis revealed that the impact of NO_2_ on life expectancy was significant in most areas, with the exception of Yunnan, Guangxi, and Hainan, where the relationship did not reach statistical significance. A few factors might be responsible for this lack of significance. Firstly, these areas generally had lower NO_2_ concentrations due to less industrial activity and urbanization, which reduced the potential impact on life expectancy. Secondly, the unique geographical and climatic conditions in these regions, such as the complex terrain in Yunnan and the humid climate in Guangxi and Hainan, might enhance pollutant dispersion and mitigate the health risks associated with NO_2_ (26)_._

**Figure 8:**
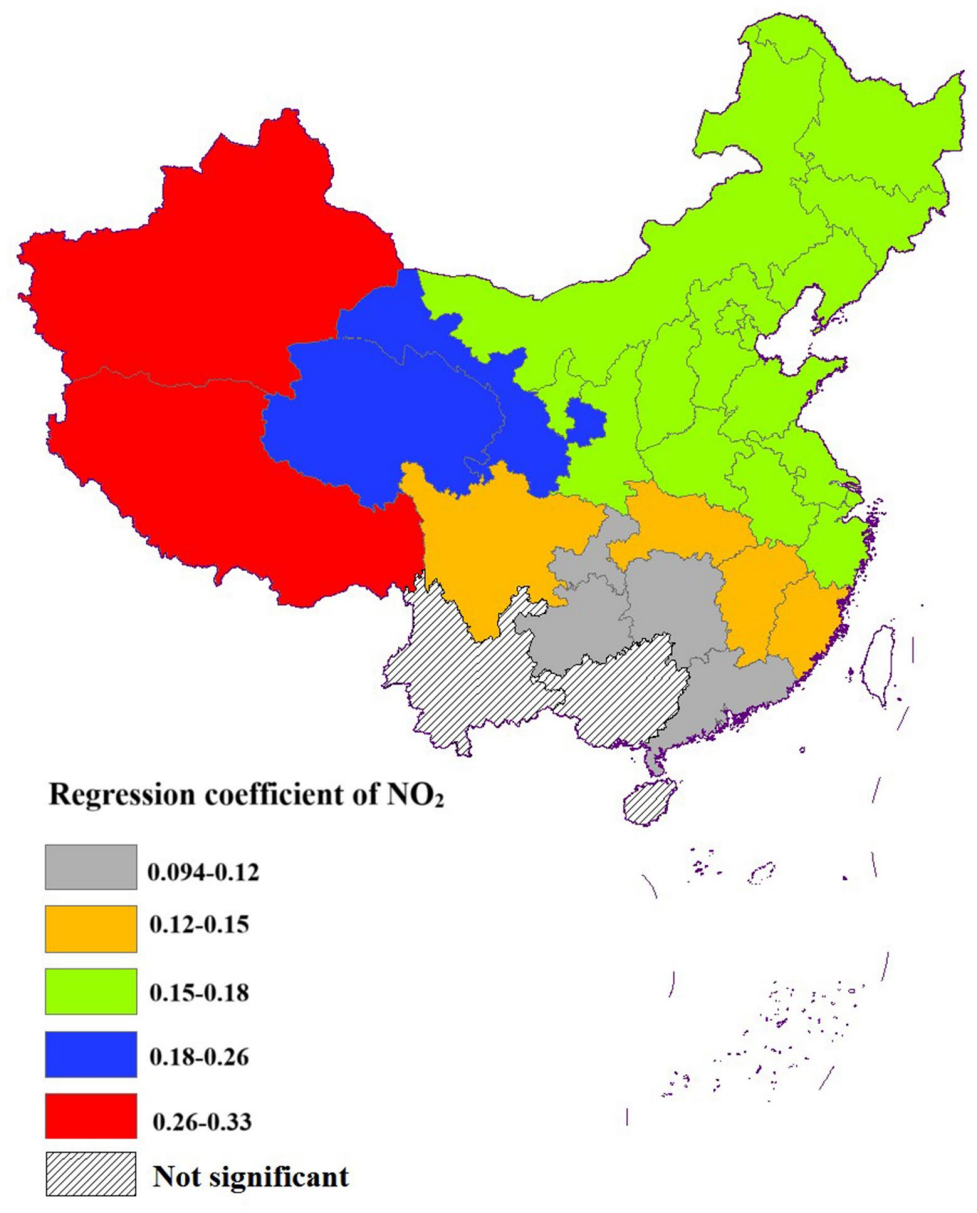
Spatial distribution of the effect of NO_2_ on life expectancy.

The spatial distribution of NO_2_ regression coefficients exhibited a decreasing gradient from the northwest to the southeast. In particular, NO_2_ had a relatively minor impact on life expectancy in most cities across the southern region (Figure 8).

##### 3.3.2.3 Impact of PM_10_ on life expectancy

**Figure 9:**
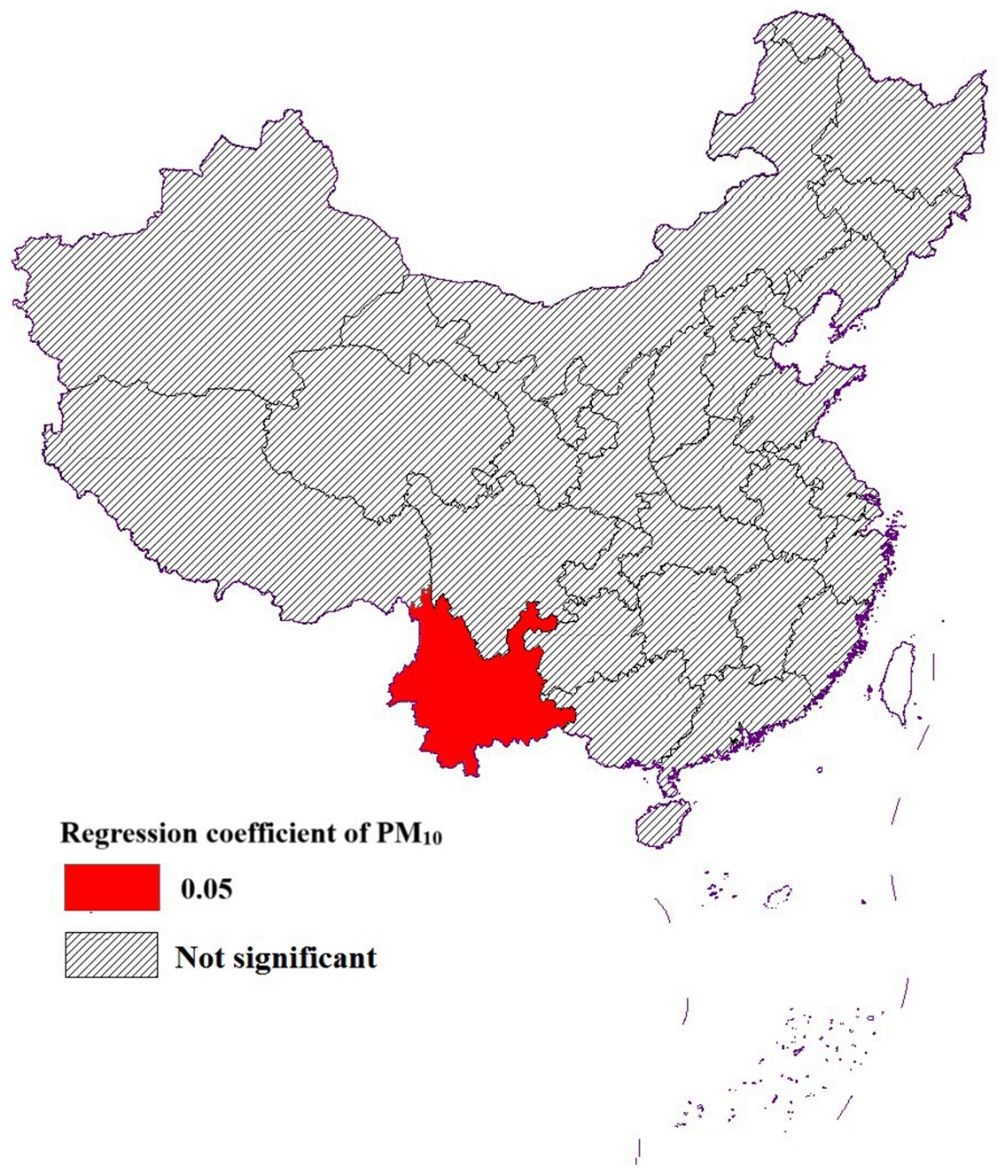
Spatial distribution of the effect of PM_10_ on life expectancy.

PM_10_ had an effect on life expectancy only in Yunnan Province, and the effect on other regions was not statistically significant. Several factors might contribute to this lack of significant. Firstly, PM_10_ concentrations in regions outside Yunnan might be relatively higher or more variable, making it difficult to detect a clear correlation with life expectancy (27). Additionally, the unique geographical and climatic conditions in Yunnan, such as its complex terrain and diverse weather patterns, could influence the dispersion and accumulation of PM_10_, leading to more pronounced health impacts (28). In contrast, other regions might have implemented more effective air quality control measures, reducing the overall impact of PM_10_ on public health (Figure 9).

#### 3.3.3 Evaluation of model fitting effectiveness

In the GWR model, the condition numbers for all provinces were consistently below 30, confirming the absence of local multicollinearity within the model. As illustrated in Table 2, among the three examined factors, only SO_2_ and NO_2_ exhibited statistically significant impacts on life expectancy in the MLR model, a finding that diverged somewhat from the GWR model results.

**Table 2:** MLR model results.

Factors	Regression coefficient	*p*	VIF
Intercept	76.49	0.000	/
SO2	−0.225	0.033	1.15
NO2	0.195	0.000	1.53
PM_10_	−0.022	0.306	1.70

**Figure 10:**
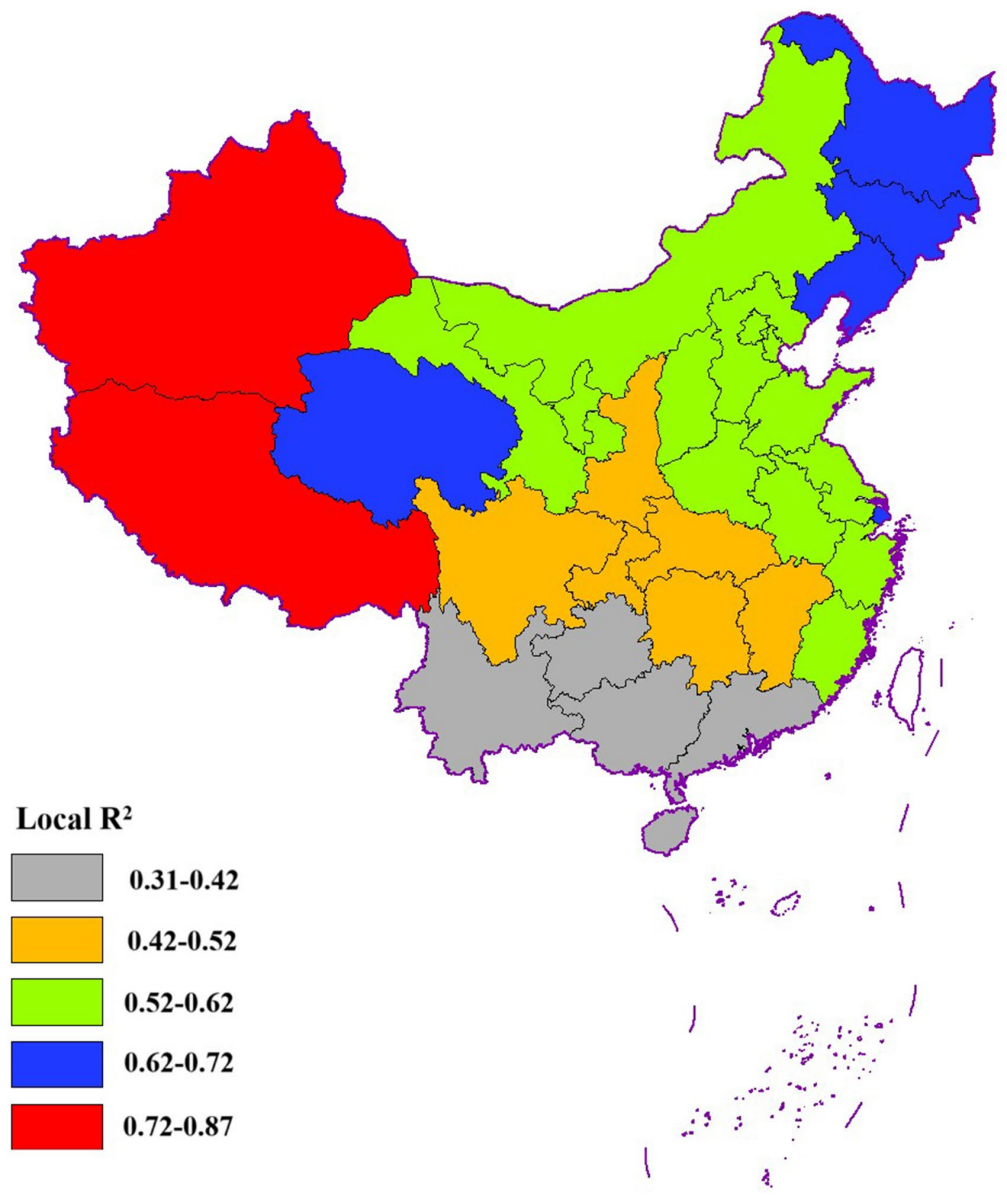
The spatial distribution of local R^2^.

Additionally, the Moran’s I index for the residuals of the MLR model was 0.40 (*p* = 0.000), whereas for the GWR model, it was 0.026 (*p* = 0.61), with a *p*-value exceeding 0.05. This indicated that the residual term displayed no spatial autocorrelation and adhered to a random distribution pattern, suggesting that the GWR model effectively captures the spatial relationships between the dependent and independent variables, thereby outperforming the MLR model in this aspect. Furthermore, as shown in Table 3, the AIC_c_ value of the GWR model was lower than that of the MLR model, and both the R^2^ and Adjusted R^2^ values were higher, underscoring the superior fitting capability of the GWR model. The local R^2^ values (Figure 10) revealed that the model fit was particularly robust in the western and northeastern regions of China, where R^2^ values were notably higher compared to other regions.

**Table 3:** Comparison of multiple linear regression model and GWR model fit.

Parametric	MLR	GWR
*AIC_c_*	130.99	112.72
*R*^2^ (%)	44.88	84.32
Adjust *R^2^* (%)	38.75	75.57

## 4 Discussion

Life expectancy serves as a robust and representative indicator of regional health status, capturing the cumulative impact of various socio-economic, environmental, and healthcare factors. This study employed the GWR model to investigate the spatial heterogeneity of air pollution factors influencing life expectancy at the provincial scale, providing insights into localized variations and their implications for public health policy.

The impact of SO_2_ on life expectancy exhibited a significant negative correlation, indicating that regions with higher SO_2_ concentrations tend to experience lower life expectancy. Notably, the detrimental effect of SO_2_ on life expectancy was more pronounced in southern cities compared to their eastern and northern counterparts. Despite the relatively lower SO_2_ concentrations in southern regions, such as Yunnan, the combined influence of unique geographical features, climatic conditions, and industrial structures might amplify the adverse effects of SO_2_ on local residents’ life expectancy. For instance, a relatively large amount of sulfur dioxide in the surroundings seeped into the indoor environment (29), and the local population might exhibit heightened sensitivity to pollutants like SO_2_. Furthermore, the relatively limited access to medical and educational resources in these southern cities might result in reduced awareness and capacity among residents to mitigate the effects of environmental pollution, thereby exacerbating the impact of SO_2_ on life expectancy (27, 30). In contrast, eastern and northern cities, characterized by more developed economies, diversified industrial structures, comprehensive environmental protection measures, and greater public awareness and ability to counteract environmental pollution, experienced a comparatively smaller impact of SO_2_ on life expectancy (31).

The analysis revealed a paradoxical positive correlation between NO_2_ concentrations and life expectancy, suggesting that regions with higher NO_2_ levels tend to exhibit longer life expectancy. This counterintuitive phenomenon might be attributed to the complex interplay between NO_2_ sources and regional socio-economic factors. In urban areas, where vehicle emissions constitute the primary source of NO_2_ (32, 33), the presence of developed transportation infrastructure often coincided with higher economic development, superior healthcare resources, and improved living standards. These factors might collectively mitigate the adverse health effects of NO_2_ exposure. Furthermore, regions with elevated NO_2_ concentrations might implement more stringent environmental protection measures, potentially reducing emissions of other harmful pollutants and indirectly benefiting public health. In contrast, economically disadvantaged areas with lower NO_2_ concentrations might suffer from overall poor environmental quality and inadequate healthcare resources, negating any potential benefits of reduced NO_2_ exposure (34). However, it was imperative to emphasize that this observed correlation did not imply a beneficial effect of NO_2_ on health. Substantial evidence from epidemiological studies demonstrated that NO_2_ exposure was associated with increased risks of cardiovascular diseases, respiratory disorders, and all-cause mortality (35). Therefore, while certain regions might experience higher life expectancy due to favorable socio-economic conditions, these benefits did not outweigh the well-documented detrimental health effects of NO_2_ exposure.

The impact of nitrogen dioxide (NO_2_) on life expectancy exhibited notable regional disparities in China, with a more pronounced effect in the northwest compared to the southeast. This spatial heterogeneity could be attributed to several factors. In northwestern regions, the delayed industrialization and urbanization processes had resulted in industrial emissions being concentrated in specific industrial parks, leading to elevated local NO_2_ concentrations and more severe health impacts on surrounding populations. Additionally, the arid climate and poor atmospheric diffusion conditions in the northwest facilitated the accumulation of pollutants near the ground, exacerbating the adverse health effects of NO_2_. In contrast, southeastern regions, characterized by developed economies, high urbanization levels, and dense transportation networks, had implemented stricter environmental protection measures and possess more robust medical resources and health protection systems. These factors had collectively mitigated the negative impact of NO_2_ on life expectancy to a considerable extent (36). Furthermore, the humid climate and superior atmospheric diffusion conditions in the southeast reduced the likelihood of prolonged pollutant retention, thereby diminishing the health risks associated with NO_2._ The regional differences in the impact of NO_2_ on life expectancy were not solely determined by pollutant concentrations and sources but were also influenced by a complex interplay of economic development, environmental policies, and climatic conditions. This underscored the importance of region-specific strategies in addressing air pollution and its health implications (37).

The analysis revealed that PM_10_ exhibited a notable impact on life expectancy exclusively in Yunnan Province, while SO_2_ also demonstrated a significant influence in the region. These findings underscored the urgent need for Yunnan Province to implement targeted pollution control measures, prioritizing the reduction of PM_10_ and SO_2_ levels to mitigate their adverse health effects.

## 5 Conclusion

In conclusion, this study revealed distinct spatial disparities and autocorrelation of life expectancy across China, highlighting the heterogeneous impacts of air pollution factors on population health. Among the selected pollutants, SO_2,_ NO_2_, and PM_10_ exhibited significant but geographically varying effects on life expectancy. Specifically, SO_2_ demonstrated a more pronounced impact in southern cities, while NO_2_ showed stronger effects in the northwestern region. Notably, PM_10_ influence was exclusively observed in Yunnan Province. These regional disparities underscored the necessity for localized air pollution control strategies.

The findings of this study provide critical insights for policymakers aiming to improve public health through air pollution mitigation. Southern provinces should prioritize SO_2_ emission reduction, whereas northwestern regions should focus on NO_2_ pollution control. For Yunnan Province, a dual approach targeting both SO_2_ and PM_10_ is recommended. Furthermore, given the cross-regional nature of air pollution, inter-provincial collaboration in developing joint prevention and control measures is crucial for effectively enhancing life expectancy across China. Policymakers should also consider integrating health impact assessments into air quality management plans to ensure that pollution control strategies are aligned with public health goals. Additionally, public awareness campaigns and stricter enforcement of environmental regulations could further support these efforts.

While this study provides valuable insights, several limitations should be acknowledged. First, the analysis relied on aggregated data at the provincial level, which may mask finer-scale variations in air pollution exposure and health outcomes. Second, the study focused on a limited set of air pollutants (SO_2_, NO_2_, and PM_10_), and future research could include other pollutants such as ozone (O_3_) to provide a more comprehensive understanding. Third, the cross-sectional design of the study limits our ability to infer causal relationships between air pollution and life expectancy. Longitudinal studies are needed to better understand the temporal dynamics of these relationships. Finally, unmeasured confounding factors, such as socioeconomic status and healthcare access, may have influenced the results, highlighting the need for more nuanced data in future analyses (38).Despite these limitations, this study contributes to the growing body of evidence on the spatial heterogeneity of air pollution’s health impacts and provides a foundation for targeted policy interventions to improve population health in China.

## Data Availability

The datasets presented in this study can be found in online repositories. The names of the repository/repositories and accession number(s) can be found in the article/Supplementary material.
